# Protein Kinase C Beta in the Tumor Microenvironment Promotes Mammary Tumorigenesis

**DOI:** 10.3389/fonc.2014.00087

**Published:** 2014-04-23

**Authors:** Julie A. Wallace, Jason R. Pitarresi, Nandini Sharma, Marilly Palettas, Maria C. Cuitiño, Steven T. Sizemore, Lianbo Yu, Allen Sanderlin, Thomas J. Rosol, Kamal D. Mehta, Gina M. Sizemore, Michael C. Ostrowski

**Affiliations:** ^1^Department of Molecular and Cellular Biochemistry, College of Medicine, The Ohio State University, Columbus, OH, USA; ^2^Comprehensive Cancer Center, The Ohio State University, Columbus, OH, USA; ^3^Department of Radiation Oncology, The Ohio State University, Columbus, OH, USA; ^4^Center for Biostatistics, The Ohio State University, Columbus, OH, USA; ^5^Department of Veterinary Clinical Sciences, College of Veterinary Medicine, The Ohio State University, Columbus, OH, USA

**Keywords:** protein kinase C beta, breast cancer, mammary neoplasms (experimental), tumor microenvironment, stroma, fibroblasts

## Abstract

Protein kinase C beta (PKCβ) expression in breast cancer is associated with a more aggressive tumor phenotype, yet the mechanism for how PKCβ is pro-tumorigenic in this disease is still unclear. Interestingly, while it is known that PKCβ mediates angiogenesis, immunity, fibroblast function and adipogenesis, all components of the mammary tumor microenvironment (TME), no study to date has investigated whether stromal PKCβ is functionally relevant in breast cancer. Herein, we evaluate mouse mammary tumor virus–polyoma middle T-antigen (*MMTV–PyMT*) induced mammary tumorigenesis in the presence and absence of PKCβ. We utilize two model systems: one where PKCβ is deleted in both the epithelial and stromal compartments to test the global requirement for PKCβ on tumor formation, and second, where PKCβ is deleted only in the stromal compartment to test its role in the TME. *MMTV–PyMT* mice globally lacking PKCβ live longer and develop smaller tumors with decreased proliferation and decreased macrophage infiltration. Similarly, when PKCβ is null exclusively in the stroma, *PyMT*-driven B6 cells form smaller tumors with diminished collagen deposition. These experiments reveal for the first time a tumor promoting role for stromal PKCβ in *MMTV–PyMT* tumorigenesis. In corroboration with these results, PKCβ mRNA (*Prkcb*) is increased in fibroblasts isolated from *MMTV–PyMT* tumors. These data were confirmed in a breast cancer patient cohort. Combined these data suggest the continued investigation of PKCβ in the mammary TME is necessary to elucidate how to effectively target this signaling pathway in breast cancer.

## Introduction

A role for protein kinase C (PKC) in cancer has been known for over 20 years when it was first recognized that phorbol esters promoted tumor formation through activation of the PKC family [reviewed in Ref. (1)]. There are 10 isozymes that make up this family of serine–threonine kinases, which is classified into three major groups by their mode of activation: the classical PKCs (PKCα, PKCβI, PKCβII, and PKCγ) are calcium and diacylglycerol (DAG) dependent; the novel PKCs (PKCδ, PKCε, PKCη, and PKCθ) are activated by DAG; and the atypical PKCs (PKCξ and PKCι) are independent of calcium and DAG. PKCβ is comprised of two splice variants, PKCβI and PKCβII, which are from the same gene (*PRKCB*) and differ only in their last 50 amino acids (2, 3). Differential PKC expression has been documented in essentially every histological cancer type including carcinomas (e.g., breast, colorectal), sarcomas (e.g., glioma), lymphomas (e.g., diffuse large B-cell lymphoma), and leukemias (e.g., B-cell chronic lymphocytic leukemia) [reviewed in Ref. (1, 4)]. Functionally, PKCs mediate various physiological processes such as proliferation, differentiation, apoptosis, cellular motility, and angiogenesis (1, 4).

Relevance for PKC in breast cancer was first reported in 1986 when Borner and colleagues observed that PKC is increased in more aggressive estrogen receptor-α (ERα) negative mammary tumor cells compared to their less aggressive ERα positive counterparts (5). This putative tumor promoting role was supported shortly thereafter by evidence of increased PKC enzymatic activity in breast tumor versus normal patient samples (6, 7). Evaluation of the specific PKC isozymes soon followed and PKCα (8–14), PKCβ (as discussed below), PKCδ (15, 16), PKCε (17), and PKCη (18, 19) were all found to have roles in breast cancer progression. Investigation of PKCβ, specifically, has revealed that both splice variants (PKCβI and PKCβII) are upregulated in breast tumor versus matched normal patient tissue (20), with cytoplasmic PKCβII associating with Ki-67 expression and HER2 positivity (21). Functionally, ectopic overexpression of either wild type (WT) or constitutively active PKCβI or PKCβII increases breast cancer cell line growth *in vitro* through upregulation of cyclin D1, while inhibition of kinase activity decreases growth as seen via introduction of dominant negative PKCβI or PKCβII, and through treatment with LY379196, a PKCβ selective inhibitor (22). Interestingly, even though PKCβII is associated with increased proliferation and highly aggressive HER2 positive disease, its expression does not correlate with patient survival in this cohort (21). An independent study also observed that PKCβ mRNA (*PRKCB*) correlates with increased grade and triple negative (ERα−/PR−/HER2−) disease, but not relapse-free survival (23).

This discrepancy between the *in vitro* breast cancer cell line and patient survival data may be due to PKCβ-mediating physiological processes within the breast tumor microenvironment (TME), which could cause the evaluation of PKCβ expression in whole tumor tissue, which contains both tumor and stroma, to be misleading. All epithelial-derived tumors (i.e., carcinomas) are surrounded by a complex TME that contains extracellular matrix proteins, endothelial cells, immune cells, and fibroblasts [reviewed in Ref. (24)]. Importantly, PKCβ function in non-epithelial cell types has been described in several model systems. Within the endothelial compartment of the TME, PKCβ is a well-known downstream effector of vascular endothelial growth factor (VEGF) signaling, which mediates angiogenesis (25–27). PKCβ is also involved in immunity through activation of NF-κB (28–30). Less is known about PKCβ specifically in tumor-associated fibroblasts, but it was recently shown that PKC is required for pancreatic cancer-associated fibroblast (CAF) invasiveness (31) as well as resistance to irradiation-induced apoptosis in primary human fibroblasts (32). PKCβ is, however, required for extracellular matrix and collagen deposition in rodent models of diabetes (33, 34). The breast TME also includes adipocytes and PKCβ null mice have decreased adipose tissue with altered lipid metabolism (35, 36). Combined, these studies suggest important roles for PKCβ in the breast TME and the need for further elucidation of PKCβ’s roles in this context.

Herein, we utilized PKCβ genomic knockout mice (37) to evaluate mammary tumorigenesis through the use of two model systems: one where PKCβ is deleted in both the mammary epithelium and the TME, and one where PKCβ is deleted only in the TME. We reveal for the first time a requirement for PKCβ in *MMTV–PyMT* (mouse mammary tumor virus–polyoma middle T-antigen) induced mammary tumor growth. *MMTV–PyMT* mice lacking PKCβ (*Prkcb*^−/−^) in both epithelial and stromal compartments have increased tumor latency with a decrease in tumor load and tumor volume. Decreased tumorigenesis in the *MMTV–PyMT*; *Prkcb*^−/−^ mice is accompanied by diminished tumor proliferation and macrophage infiltration with angiogenesis seemingly unaffected. To test directly whether PKCβ in the TME is functionally relevant, we orthotopically injected B6 *PyMT* tumor cells, a *MMTV–PyMT* derived mammary tumor cell line (38), into WT or *Prkcb*^−/−^ mice. Tumor volume was similarly decreased in the absence of stromal PKCβ confirming a requirement for PKCβ in the mammary cancer TME. Most interestingly, collagen deposition was decreased in this model. Moreover, fibroblasts isolated from the *MMTV–PyMT* tumors have dramatically higher levels of PKCβII mRNA (*Prkcb*) than WT mammary fibroblasts, whereas *Prkcb* in the *MMTV–PyMT* epithelium is decreased. This increase in stromal *PRKCB* is similarly observed in a human breast cancer patient cohort confirming the translational relevance of PKCβ function in the TME.

## Materials and Methods

### Animal breeding

All animal procedures were approved by The Ohio State University Institutional Animal Care and Use Committee (protocol #2007A0120-R2). *Prkcb*^−/−^ (35, 37) and *MMTV–PyMT* mice (39) have been described. The *MMTV–PyMT* mice were bred into C57Bl/6 at least 10 generations. *MMTV–PyMT* males were bred with *Prkcb*^+/−^ females to generate *MMTV–PyMT*; *Prkcb*^+/−^ male progeny. The *MMTV–PyMT*; *Prkcb*^+/−^ males were then bred with *Prkcb*^+/−^ females to generate *MMTV–PyMT*; *Prkcb*^+^*^/^*^+^ and *MMTV–PyMT*; *Prkcb*^−/−^female progeny for tumor analysis. Genotyping primers and conditions for the *Prkcb*^−/−^ mice have been previously reported (35). Genotyping primers and conditions for the *MMTV–PyMT* mice are described on The Jackson Laboratory website.

### Tumor studies and fibroblast/epithelial isolation

*MMTV–PyMT*; *Prkcb*^+^*^/^*^+^ and *MMTV–PyMT*; *Prkcb*^−/−^mice were palpated starting at 3 months of age. Upon detection of palpable tumors, tumorigenesis was allowed to progress three additional weeks, upon which the tumors were harvested. At the time of harvest, tumor burden was measured as a percentage of the tumor weight relative to total body weight, and total tumor volume was calculated by caliper measurements (length × width × height). Each tumor was then either frozen or fixed in formalin for later histological analysis. For the orthotopic injection study, we injected 3 × 10^6^ B6 *PyMT* tumor cells into the inguinal mammary fat pads of either *Prkcb*^+^*^/^*^+^ or *Prkcb*^−/−^mice. Tumors were monitored by weekly palpation. When the largest tumor reached 1 cm in diameter, a final measurement was determined by caliper and all tumors were harvested. B6 *PyMT* cells were a generous gift from Tsonwin Hai and have been described previously (38). B6 *PyMT* cells were maintained *in vitro* using DMEM/F-12 plus 10% fetal bovine serum plus penicillin/streptomycin. Isolation of mammary fibroblasts and epithelium was performed by gravity separation as has been described (40, 41).

### Immunofluorescence and histological staining

Tumor tissue was fixed in formalin for 24 h, transferred to 70% ethanol, paraffin embedded, and sectioned (5 μm). Sections were rehydrated through xylenes and an ethanol series. Antigen retrieval was performed using DAKO antigen retrieval in a steamer for 30 min and sections were blocked with DAKO protein block. Primary detection of F4/80 (Molecular Probes), Ki-67 (DAKO), and MECA-32 (BD Pharmingen), was performed overnight at 4°C. Alexafluor-488 and Alexafluor-596 (Invitrogen) were used for secondary detection and DAPI as a nuclear counterstain. To quantify, five images (20× fields) were taken per mouse. Quantification of F4/80 and Ki-67 staining was done using the count tool in Adobe Photoshop CS5. Percent positivity of each stain was determined relative to the total number of cells as visualized by the DAPI counterstain. Quantification of MECA-32 staining was measured as percent positive area using Image J (42). Hematoxylin and eosin (H&E) and Masson’s Trichrome staining was performed by the Solid Tumor Pathology Core at OSU.

### RNA isolation and quantitative real-time PCR

Total RNA was isolated using Trizol reagent (Invitrogen), treated with Turbo DNase I (Ambion) and cDNA generated using Superscript III Reverse Transcriptase (Invitrogen), all per manufacturer’s recommendations. *Prkcb* (F = gaaactcgaacgcaaggaga; R = accg gtcgaagttttcagc; Probe #83) and *Rpl4* (F = gatgagctgtatggcacttgg; R = cttgtgcatgggcaggtta; Probe #38) were detected using the Roche Universal Probe Library system.

### Dataset and statistical analyses

The Karnoub breast cDNA microarray dataset of human breast tumors was retrieved from Oncomine (oncomine.org) for *in silico* analyses (43). *PRKCB* expression was compared between invasive ductal breast carcinoma samples versus normal breast tissue. Statistical significance was determined by Student’s *t*-test assuming a two-tailed distribution and equal variance. Kaplan–Meier survival curves were generated and statistical significance determined using log-rank. Tumor volume measurements and genetic marker staining was compared between two genetic groups. Log transformation of the data was performed if needed. Two-sample *t*-test or non-parametric Mann–Whitney test was applied after normality check. Type I error level was controlled at 0.05 level, and adjusted by Bonferroni’s method when multiple comparisons were made.

## Results

### Global PKCβ deletion reduces mammary tumorigenesis

**Figure 1:**
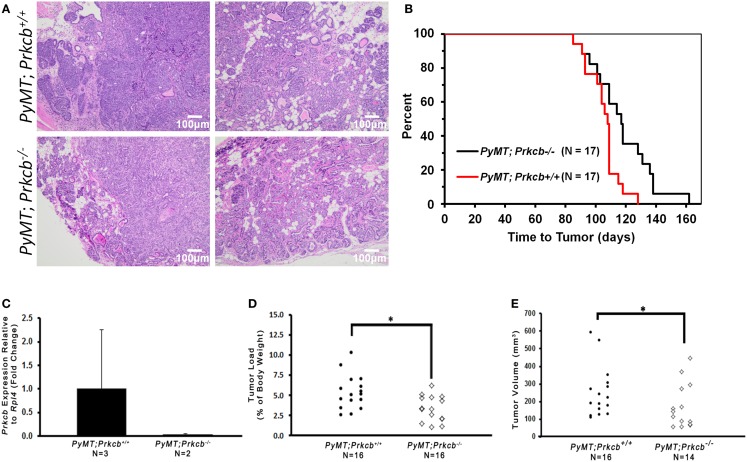
**Global PKCβ deletion reduces mammary tumorigenesis.** **(A)** Representative H&E stained mammary tumors from *PyMT; Prkcb*^+^*^/^*^+^ and *PyMT; Prkcb*^−/−^mice. Scale bar, 100 μm. **(B)** Kaplan–Meier curve comparing overall tumor-free survival of *PyMT; Prkcb*^+^*^/^*^+^ and *PyMT; Prkcb*^−/−^mice (*n* = 17 per group, *P < 0.02 obtained by log-rank). **(C)** *Prkcb* expression in mammary tumor cells of *PyMT; Prkcb*^+^*^/^*^+^ and *PyMT; Prkcb*^−/−^mice normalized to *Rpl4* (*n* = 3 and 2, respectively, bars represent means + standard deviation). **(D)** Dissected tumor load (percentage of tumor mass relative to total body mass) from *PyMT; Prkcb*^+^*^/^*^+^ and *PyMT; Prkcb*^−/−^mice (*n* = 16 per group, *P < 0.05 obtained from Mann–Whitney test). **(E)** Dissected tumor volume (mm^3^) isolated from *PyMT; Prkcb*^+^*^/^*^+^ and *PyMT; Prkcb*^−/−^mice (*n* = 16 and 14, respectively, *P < 0.05 obtained from two-sample *t*-test of log based 10 transformation applied on original right skewed data).

Members of the PKC family are known to influence proliferation, differentiation, apoptosis, cellular motility, and angiogenesis (1, 4), leading us to hypothesize that loss of PKCβ would cause decreased mammary tumorigenesis in the *MMTV–PyMT* mammary tumor model. To evaluate the role for PKCβ in tumorigenesis, we bred the *Prkcb*^−/−^ knockout mice with those harboring the *MMTV*–*PyMT* transgene, a well-characterized murine model of mammary tumor formation (44). We monitored tumor development and harvested tumors 3 weeks after initial palpation. Although the tumors between the two groups looked histologically similar by hematoxylin and eosin (H&E) staining (Figure 1A), *MMTV–PyMT* mice lacking PKCβ (*MMTV–PyMT; Prkcb*^−/−^) developed tumors at a longer latency when compared to control *MMTV–PyMT*; *Prkcb*^+^*^/^*^+^ mice (Figure 1B). The absence of *Prkcb* in the tumors was confirmed (Figure 1C). Not only did the absence of PKCβ delay tumor onset, but the tumors that developed in the *MMTV–PyMT*; *Prkcb*^−/−^ mice were smaller as measured by both tumor load (Figure 1D) as well as total volume (Figure 1E). These data indicate that the global absence of PKCβ in this mouse model has a measurable impact on mammary tumorigenesis.

### Global PKCβ deletion decreases tumor cell proliferation and macrophage recruitment, but not tumor vasculature

**Figure 2:**
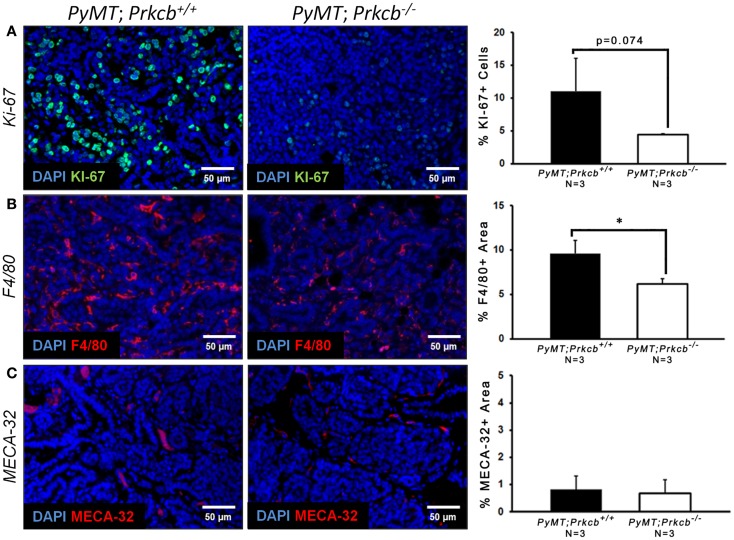
**Global PKCβ deletion decreases tumor cell proliferation and macrophage recruitment, but not tumor vasculature.** **(A)** Left: representative images of Ki-67 staining of proliferating cells within *PyMT; Prkcb*^+^*^/^*^+^ and *PyMT; Prkcb*^−/−^tumors *in vivo*. Scale bar, 50 μm. Right: graph of percentage of Ki-67+ cells relative to total DAPI+ cells (*n* = 3 per group, bars represent means + standard deviation, *P = 0.074 obtained from two-sample *t*-test). **(B)** Left: representative images of F4/80 staining of macrophage cells within *PyMT; Prkcb*^+^*^/^*^+^ and *PyMT; Prkcb*^−/−^tumors *in vivo*. Scale bar, 50 μm. Right: graph of percentage of F4/80+ area relative to total area of image (*n* = 3 per group, bars represent means + standard deviation, *P < 0.05 obtained from two-sample *t*-test). **(C)** Left: representative images of MECA-32 staining of tumor vasculature within *PyMT; Prkcb*^+^*^/^*^+^ and *PyMT; Prkcb*^−/−^tumors *in vivo*. Scale bar, 50 μm. Right: graph of percentage of MECA-32+ area relative to total area of image (*n* = 3 per group, bars represent means + standard deviation, *P* > 0.05 obtained from two-sample *t*-test).

To explain the delayed onset and decreased tumor size in mice lacking PKCβ, we stained for a proliferation marker (Ki-67) within the *PyMT* tumors of both genetic groups. We saw an observable decrease in the percentage of Ki-67 positive cells in *MMTV–PyMT*; *Prkcb*^−/−^ mice (Figure 2A), indicating that PKCβ influences *PyMT* tumor progression by modulating tumor cell proliferation capacity. Given the recent knowledge that the TME can influence tumor cell proliferation (41), as well as the known function for PKCβ in endothelium and immune cells, we also investigated macrophage infiltration and tumor vasculature in the *MMTV–PyMT* tumors with and without PKCβ.We saw a significant decrease in percentage of F4/80 positive area, a macrophage marker, in *MMTV–PyMT; Prkcb*^−/−^ tumors (Figure 2B), indicating that loss of PKCβ within macrophages influences their recruitment to the tumor site. We saw no measurable difference in tumor vascularization, as measured by MECA-32 staining, between the *Prkcb*^−/−^ and WT groups (Figure 2C), demonstrating that the delayed tumor onset and growth observed in *Prkcb*^−/−^ mice was independent of vasculature formation.

### Loss of PKCβ in stromal compartments decreases tumor volume and collagen deposition, but has no effect on tumor cell proliferation, vascularization, or macrophage infiltration

**Figure 3:**
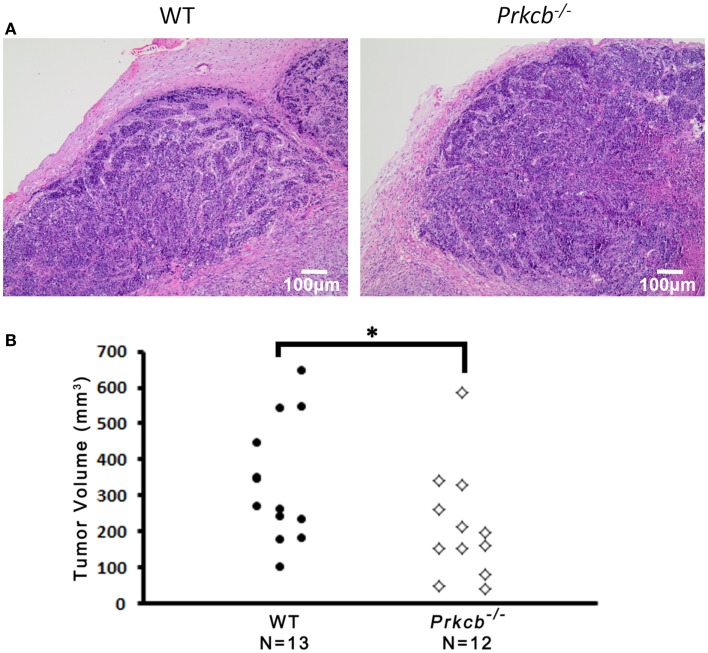
**Loss of PKCβ in stromal compartments decreases tumor volume.** **(A)** Representative H&E stained B6 *PyMT* tumors developed in wild type (WT) and *Prkcb*^−/−^ mice. Scale bar, 100 μm. **(B)** Dissected B6 *PyMT* tumor volume isolated from wild type (WT) and *Prkcb*^−/−^ mice (*n* = 13 and 12, respectively, *P < 0.05 obtained from Mann–Whitney test).

**Figure 4:**
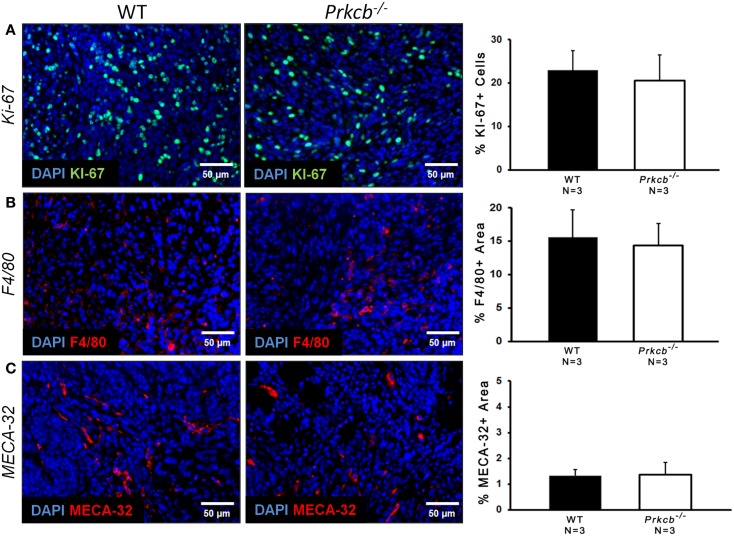
**Loss of PKCβ in stromal compartments has no effect on tumor cell proliferation, vascularization, or macrophage infiltration**. **(A)** Left: representative images of Ki-67 staining of proliferating cells within B6 *PyMT* tumors *in vivo*. Scale bar, 50 μm. Right: graph of percentage of Ki-67+ cells relative to total DAPI+ cells (*n* = 3 per group, bars represent means + standard deviation, *P* > 0.05 obtained from two-sample *t*-test). **(B)** Left: representative images of F4/80 staining of macrophage cells within B6 *PyMT* tumors *in vivo*. Scale bar, 50 μm. Right: graph of percentage of F4/80+ area relative to total area of image (*n* = 3 per group, bars represent means + standard deviation, *P* > 0.05 obtained from two-sample *t*-test). **(C)** Left: representative images of MECA-32 staining of B6 *PyMT* tumor vasculature *in vivo*. Scale bar, 50 μm. Right: graph of percentage of MECA-32+ area relative to total area of image (*n* = 3 per group, bars represent means + standard deviation, *P* > 0.05 obtained from two-sample *t*-test).

**Figure 5:**
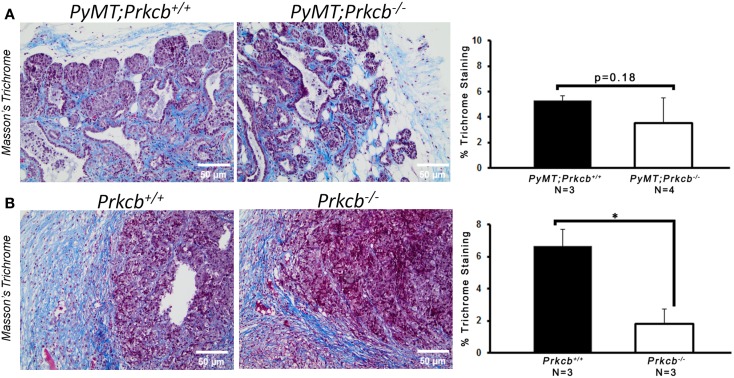
**Loss of PKCβ in stromal compartments decreases collagen deposition**. **(A)** Left: representative images of Masson’s Trichrome staining within *PyMT; Prkcb*^+^*^/^*^+^ and *PyMT; Prkcb*^−/−^tumors *in vivo*. Scale bar, 50 μm. Right: graph of percentage of Trichrome collagen (blue) staining relative to total area (*n* = 3 and 4, respectively, bars represent means + standard deviation, *P* = 0.18 obtained from two-sample *t*-test). **(B)** Left: representative images of Masson’s Trichrome staining within B6 *PyMT* tumors *in vivo*. Scale bar, 50 μm. Right: graph of percentage of Trichrome collagen (blue) staining relative to total area. *n* = 3 per group, bars represent means + standard deviation, *P* < 0.05 obtained from two-sample *t*-test).

To determine the importance of PKCβ signaling within the stroma, we orthotopically injected *Prkcb*^−/−^ and WT mice with B6 *PyMT*-derived tumor cells. As seen with the genetic model, tumors developing in *Prkcb*^−/−^ mice exhibited no obvious histological differences (Figure 3A), but did result in significantly smaller tumor volumes compared to WT controls (Figure 3B). This result, in conjunction with the results presented in Figure 1, confirms that stromal PKCβ is important in *PyMT* mammary tumor formation. In order to further assay the effect of stromal PKCβ deletion within the tumor and its microenvironment, tumors were also evaluated for histological alterations within the distinct cellular compartments. Interestingly, there was no difference in Ki-67, F4/80, or MECA-32 positivity between *Prkcb*^−/−^ and WT mice (Figure 4). Thus, losing PKCβ signaling in the stroma decreased tumor size, but had no effect on tumor cell proliferation, vascularization, or macrophage recruitment. To evaluate whether stromal fibroblasts could be responsible for the observed decrease in tumor size in *Prkcb*^−/−^ mice, we evaluated collagen deposition through Masson’s Trichrome staining. The absence of PKCβ specifically in the stroma resulted in significantly diminished collagen deposition while this decrease was not as pronounced in the global knockout model (Figure 5). These results imply that PKCβ signaling in fibroblasts is pro-tumorigenic in the mammary gland.

### PKCβ is increased in PyMT tumor-associated fibroblasts and patient breast cancer stroma

**Figure 6:**
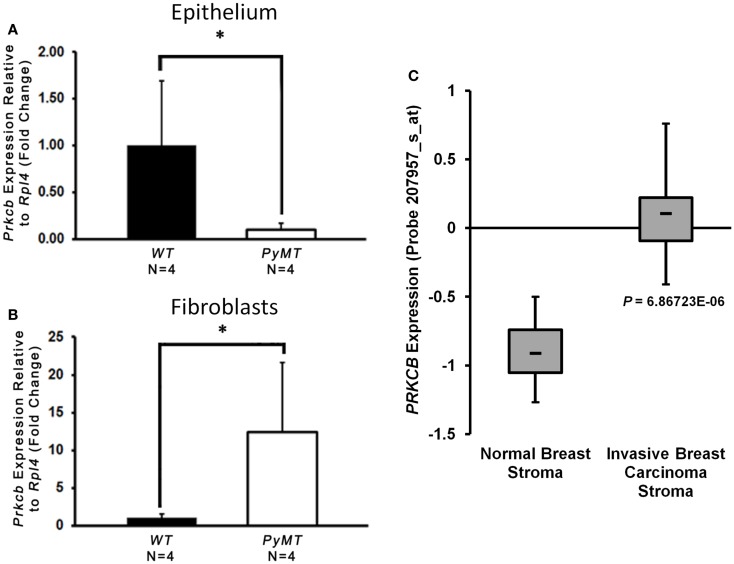
**PKCβ is increased in PyMT tumor-associated fibroblasts and patient breast cancer stroma**. **(A)** *Prkcb* expression in primary mammary fibroblasts normalized to *Rpl4* and relative to WT. Cells isolated from *PyMT* mice 2-weeks after first palpable tumor and WT littermate controls (*n* = 4, bars represent means + standard deviation, *P < 0.05 obtained from two-sample *t*-test). **(B)** *Prkcb* expression in primary mammary epithelial cells normalized to *Rpl4* and relative to WT. Cells isolated from *PyMT* mice 2-weeks after first palpable tumor and WT littermate controls (*n* = 4, bars represent means + standard deviation, *P < 0.05 obtained from two-sample *t*-test). **(C)** Box and whisker plot of *PRKCB* expression in invasive ductal breast carcinoma stroma compared to normal breast stroma in the Karnoub breast dataset (normal, *n* = 15; IDC, *n* = 7, *P < 10^−5^).

Given the decreased collagen deposition in the absence of stromal PKCβ as just discussed, in addition to previously described roles for PKCβ in fibroblast function (31–34), we further hypothesized that *MMTV–PyMT* tumorigenesis may be, at least in part, due to aberrant PKCβ expression in the mammary fibroblasts. To this end, we isolated primary mammary tumor epithelial cells and tumor-associated fibroblasts from *PyMT* mice 2 weeks after palpable tumor formation. We also isolated mammary epithelial cells and fibroblasts from age-matched WT littermate controls. We assayed the expression level of *Prkcb* mRNA in both cellular compartments by quantitative real-time PCR (qRT-PCR), and observed a significant decrease in *Prkcb* expression in mammary epithelial cells derived from *PyMT* tumors (Figure 6A), possibly indicating that epithelial PKCβ is tumor suppressive during *PyMT*-driven tumor formation. In contrast, a dramatic increase in *Prkcb* mRNA in *PyMT* tumor-associated fibroblasts was observed compared to WT mammary fibroblasts (Figure 6B). These data, in combination with the decreased tumor formation we observed in *Prkcb*^−/−^ mice, suggest a pro-tumorigenic role for PKCβ in mammary tumor fibroblasts. To confirm these findings in human disease, we evaluated *PRKCB* in a publicly available breast cancer dataset [Karnoub et al. (43)] comparing stroma isolated from invasive ductal breast carcinoma compared to normal breast tissue. In this dataset, *PRKCB* is significantly increased in breast cancer stroma versus normal confirming the translational relevance of PKCβ in the breast TME (Figure 6C).

## Discussion

The PKC family is well-documented to have a variety of roles in cancer [reviewed in Ref. (1, 4)] with PKCβ functioning specifically in a number of cancer types including in breast (20–23, 45). Prior evaluation of PKCβ in breast cancer has utilized overexpression studies *in vitro* (20, 45) and expression analyses in patient tumor tissue: both by immunohistochemistry of tumor sections (21) and through whole tumor extraction (20, 23). While the *in vitro* breast cancer cell line data are highly suggestive of a tumor promoting role for PKCβ in breast cancer, tumor expression at both RNA (23) and protein (21) levels does not correlate with breast cancer patient survival. Given that PKCβ is required for VEGF-induced angiogenesis (25–27), immunity via NF-κB signaling (28–30), pro-tumorigenic fibroblast properties (31, 32), and adipocyte lipid metabolism (35, 36), it is likely that our understanding of how PKCβ functions in breast tumorigenesis requires further evaluation of its role in the breast TME.

In this study, we took advantage of the genomic *Prkcb*^−/−^ mouse (37). These mice are viable allowing for investigation of mammary tumorigenesis in the absence of PKCβ either throughout the whole animal as we investigated in *MMTV–PyMT* mice with and without PKCβ, or through transplantation approaches where we injected a *MMTV–PyMT* derived tumor cell line (B6 *PyMT* cells) into either WT or PKCβ null mammary fat pads. The *Prkcb*^−/−^ mice were crossed with the *MMTV–PyMT* mouse model of breast cancer, which is a valuable translational tool given that tumor formation in these mice recapitulates disease progression as seen in humans (44). Furthermore, the importance of the mammary TME in *MMTV–PyMT*-induced tumorigenesis has been well-described. Alterations in signaling from the endothelium (46), fibroblasts (41, 47, 48), immune cells (49–52), and adipocytes (53–55) have all been shown to alter tumor growth and/or metastatic progression in these mice. Moreover, increased stromal collagen deposition hastens *MMTV–PyMT* tumor development and metastatic spread (56).

As described herein, *MMTV–PyMT*; *Prkcb*^−/−^ mice develop smaller tumors at a longer latency than their *MMTV–PyMT*; *Prkcb*^+^*^/^*^+^ counterparts. This decrease in tumorigenesis is associated with a reduction in proliferation and macrophage recruitment. Tumor-associated macrophages in breast cancer act in a pro-tumor manner by upregulation of M2 functions such as promoting angiogenesis, remodeling of the tumor matrix, and suppressing the adaptive immune response (57, 58). Furthermore, M2 macrophages are thought to have an immunomodulatory function that activates tumor cell proliferation (59) suggesting a possible mechanistic connection between the observed differences in proliferation and macrophage number upon loss of PKCβ. Surprisingly, even with the well-described role for PKCβ in VEGF signaling (25–27), there was no alteration in the tumor vasculature in PKCβ null *PyMT* mice. Similar findings were observed in the injection model. However, in this case we saw no difference in proliferation, macrophage recruitment, or angiogenesis, but did see a decrease in collagen deposition. These results imply a mechanistic role for PKCβ signaling in fibroblast activity, specifically in the synthesis of collagen. It is important to note that this model only evaluates tumor progression, not initiation, since the B6 *PyMT* cells are already transformed. Future studies are required using *Prkcb* floxed mice to conditionally delete PKCβ in the endothelial (*Tie2-cre*), macrophage (*Lys-cre*), and fibroblast (*Fsp-cre*) specific cellular compartments. These mice can then be crossed with the *MMTV–PyMT* as well as other murine models of breast cancer to investigate how PKCβ alters mammary tumor initiation. While this investigation is well beyond the scope of the current study, it will be necessary to truly define the specific breast TME component where PKCβ has the most tumor altering effect.

Support for a pro-tumorigenic role for PKCβ in the breast TME is evidenced by the significantly upregulated expression of *Prkcb* in fibroblasts isolated from *MMTV–PyMT* mammary tumors when compared to WT mammary glands. Increased *PRKCB* in breast tumor stroma versus normal stroma was confirmed in a breast cancer patient cohort. Interestingly, we observed a significant decrease in *Prkcb* expression in the epithelial fraction of the *MMTV–PyMT* tumors. The concomitant decrease of *Prkcb* in epithelium and increase in the stroma suggests that whole tumor analysis of PKCβ either at the mRNA or protein level could be misinterpreted as showing no observable difference in expression. Likewise, observable differences may be reflective of expression changes in specific cellular populations confounding any predictive power of PKCβ. This is supported by the fact that similar to published studies (21, 23), we observed no significant correlation of *PRKCB* with patient outcome in several publicly available breast cancer datasets (data not shown). Given its role in the TME as described herein, PKCβ expression can only be truly defined through immunohistological evaluation of whole tumor sections in order to delineate tumor versus stromal expression. Pursuing whether PKCβ expression is predictive in breast cancer is critical because a number of PKCβ selective inhibitors have shown efficacy in pre-clinical and clinical trials for a multitude of cancer types (4, 60). To date, no studies have proven efficacious in breast cancer patients, but this could be due to the absence of a proper biomarker as tumor expression of PKCβ has not been evaluated in trial participants [(61, 62); clinicaltrials.gov]. Continued investigation into the mechanism of PKCβ function in breast tumor stroma through the use of the conditional knockout models as described above will undoubtedly translate to a greater understanding of how to clinically evaluate PKCβ tumor expression and ultimately use this aberrant signaling pathway as a therapeutic target.

## Conflict of Interest Statement

The authors declare that the research was conducted in the absence of any commercial or financial relationships that could be construed as a potential conflict of interest.
